# Physiological role of isocitrate lyase in dibenzo-*p*-dioxin and dibenzofuran metabolism by *Sphingomonas wittichii* RW1

**DOI:** 10.1186/s43141-022-00334-3

**Published:** 2022-03-30

**Authors:** Rayan M. Faisal, Aveen H. Rasol

**Affiliations:** 1 Department of Biology, College of Science, University of Mosul, Mosul, Iraq; 2 Department of Biochemistry and Microbiology, Rutgers University, New Brunswick, NJ USA

**Keywords:** Dibenzofuran, Dibenzo-*p*-dioxin, Gene knockout, Isocitrate lyase, *S. wittichii* RW1

## Abstract

**Background:**

*Sphingomonas wittichii* RW1 is one out of three strains capable of metabolizing dioxin as a sole source for carbon and energy. Under laboratory conditions the degradation rates for these aromatics are relatively high (5 and 8 h for dibenzofuran (DBF) and dibenzo-*p*-dioxin (DD), respectively). However, their degradation rates are much lower in the environment due to several factors. One of these factors is the availability of other carbon sources. Acetate is a metabolized carbon source by *S. wittichii* RW1 and its presence in the environment would have a negative impact on DBF and DD degradation. In addition, expression of most of the genes for DBF and DD degradation were downregulated when grown on acetate compared to their growth on DBF and DD. We hypothesized that blocking the acetate utilization pathway in *S. wittichii* RW1 would prevent it from using acetate when present along with DD and DBF in contaminated sites.

**Results:**

Blocking the glyoxylate shunt by deleting isocitrate lyase gene (*icl*) prevented the mutant strain (RW1Δicl) from using acetate as a sole carbon source thus depending on available DBF and DD in polluted sites. Our results showed that deletion of *icl* did not affect growth of *S. wittichii* RW1 on DBF and DD but blocked it from growing on acetate.

**Conclusion:**

Our results introduces an engineered strain that can be used as a new candidate to clean dioxin-contaminated sites which are rich with acetate.

## Background

*Sphingomonas wittichii* RW1 is one out of three strains capable of metabolizing dibenzo-*p*-dioxin as a sole source for carbon and energy, accordingly, has received extensive research from many scientists worldwide [3, 16]. Dioxins are a group of chemically related aromatics that include polychlorinated dibenzo-*p*-dioxins and dibenzofurans. These compounds are chemically inert, recalcitrant, lipid soluble, and toxic to humans and animals [17]. Dioxins are released to the environment as by products from the manufactory of pesticides and herbicides, incineration of wastes, or can be produced naturally from forest fires and volcanoes eruption; therefore, dioxins have become ubiquitous pollutants in the environment [21]. Reports have stated that more than 50% of the dioxins excluded to the environment is from incineration of municipal solid waste. These persistent compounds remain in the sediments for decades which is why it is necessary measure dioxin concentration if waste water effluent [19].

Degradation rates of *S. wittichii* RW1 for dibenzo-*p*-dioxin (DD) and dibenzofuran (DBF) under laboratory condition are relatively high, 5 and 8 h for DD and DBF, respectively, when grown on 5 mM of each substrate [24]. However, their degradation capabilities in the environment are restricted to nutrient availability, generation of dead end products, trace concentrations of dioxins, water activity, and low adaptation to soil environment [4, 5].

Attempts to engineer *S. wittichii* RW1 to improve its degradation capabilities have succeeded by introducing membrane superchannels from *Sphingomonas sp.* A1. These superchannels facilitated the incorporation of polyaromatic hydrocarbons into *S. wittichii* RW1 cells thus enhanced the degradation rate of hazardous compounds in contaminated sites [1]. Interestingly, genome analysis of *S. wittichii* RW1 revealed that this strain contained genes that carried the ability to perform all the steps included in the biphenyl degradation pathway except the dehydrogenation of *cis* 2,3-dihydro-2,3-dihydroxybiphenyl and this was the only missing gene that prevented it from growing on biphenyl. Engineering this strain by introducing an appropriate dehydrogenase from a biphenyl degrader enabled *S. wittichii* RW1 to grow on biphenyl at relatively high rates [8].

Acetate is an important carbon source and easy to use compound. It is both aerobically and anaerobically metabolized by bacteria. As a result, inhibiting such microbial activities leads to acetate accumulation in soil. Acetate concentrations varies according to type of soil being the highest in salt-marsh sediments that ranges between 0.1 and 1.0 mM [10]. Acetate is produced in soils by several pathways, it can be produced anaerobically by bacteria via acetogenesis or through anaerobic degradation of cellobiose [12]. Evidence for release of acetate from fine roots and associated mycorrhiza have also been detected [11]. Acetate is a preferable carbon source by *S. wittichii* RW1 and its presence in the environment would have a negative impact on DD and DBF degradation. In addition, expression of most of the genes for DD and DBF degradation is down regulated when grown on acetate compared to their growth on DD and DBF [9]. Oxygen uptake by *S. wittichii* RW1 resting cells grown on acetate and subjected to DD and DBF was relatively less compared to resting cells on DD and DBF [24].In addition, acetate has been reported to inhibit the degradation rate of some polyaromatic hydrocarbons (PAH) in the environment probably due to its preference by PAH degrading microorganisms [20]. The presence of more than one carbon source in the environment where one is more favorable than the other leads to diauxic growth where the organism cannot metabolize the unfavorable compound until the favorable one is totally consumed [13]. Therefore, we hypothesized that blocking the acetate utilization pathway in *S. wittichii* RW1 would prevent it from using acetate when present along with DD and DBF in contaminated sites. Engineered strains of *S. wittichii* RW1 will obtain all their carbon source from contaminated aromatics instead of utilizing acetate. Few studies have been conducted on improving biodegradation capabilities in *S. wittichii* RW1, however, improving biodegradation capabilities by engineering metabolic pathways has not been reported elsewhere.

## Materials and methods

### Bacterial strains and plasmids

*S. wittichii* RW1 was kindly provided by Dr. Gerben Zylstra at Rutgers University (New Jersey, USA). *Escherichia coli* DH5α was used as the recipient in transformation experiments (Invitrogen, Waltham, MA). The cloning vector pGEM-7Z(+) (Promega, Madison, Wisconsin) was used to clone *icl* gene containing fragment. The low copy number plasmid pRK415 was kindly provided by Dr. Donald Kobayashi at Rutgers University (New Jersey, USA) and used to construct the final knockout clone and deliver it to strain RW1 to promote gene disruption.

### Molecular techniques and DNA manipulations

Ultraclean microbial DNA isolation kit (MoBio, Carlsbad, CA) was used to isolate genomic DNA from *S. wittichii* RW1 by following the protocol supplied. Plasmid DNA was extracted from *E. coli* DH5α using Nucleospin plasmid (NoLid) purification kit (Macherey-Nagel, Germany) and following the recommendations provided. General molecular techniques including restriction enzyme digestion, gel electrophoresis, ligation, and transformation were performed according to Sambrook et al. [22]. DNA fragments were amplified using the Phusion high fidelity PCR (polymerase chain reaction) kit (New England Biolabs) and purified from PCR solution or 1% agarose gel when necessary by QiaexII extraction kit (Qiagen). DNA sequence of the final clone introduced in strain RW1 was sequenced to verify that it lacked undesired mutations. Sequencing was done through GENEWIZ sequencing company (New Jersey, USA).

### Culturing of *S. wittichii* RW1

In growth curve experiments, *S. wittichii* RW1 was grown in 250 ml flasks containing 50 ml MSB medium (Mineral Salts Basal medium) and incubated at 28 ^o^C with shaking (180 rpm). MSB was supplemented with acetate (10 mM), DBF (3 mM), and DD (3 mM) as sole carbon sources individually. Acetate was added as sodium acetate salt while DBF and DD were added as crystals. Growth curve experiments were conducted in triplicates and optical density readings were recorded at 2 h intervals. Growth curves were drawn using GraphPad Prism (v. 8.0.2), and all statistics including calculation of average and standard deviation were automatically calculated by Prism and shown on the growth curves.

For the purpose of searching for gene knockout strains, *S. wittichii* RW1 containing the knockout vector (shown below) was grown in 5 ml Luria-Bertani broth (LB) and incubated at 28 ^°^C. A series of transfers on the same medium was done and dilutions were spread on LB plates containing kanamycin to select for colonies that were kanamycin-resistant and tetracycline-sensitive. All bacterial strains used in this study were kept at – 80 ^°^C.

### Construction of knockout vector

A 1.76-kb DNA fragment containing isocitrate lyase gene (*icl*) was PCR amplified from the genomic DNA of *S. wittichii* RW1 using the primers icl-F (5’GGGGAATTCGGTCGAGGTCGATCATCAGCATCG 3′) and icl-R1 (5′ CCCTCTAGATTACGCCGCGAACTGGTTCATCG 3′) that introduced *Eco*RI and *Xba*I restriction sites, respectively. This fragment was cloned to the *Eco*RI and *Xba*I sites of pGEM-7Z. The resulted construct was digested with *Pst*I and a *Pst*I digested kanamycin cassette was cloned to this site to disrupt the *icl* reading frame. The fragment containing kanamycin cassette was PCR amplified using icl-F and icl-R2 (5′ GGGGAATTCTTACGCCGCGAACTGGTTCATCG 3′) that introduced a second *EcoR*I restriction site. This fragment was cloned to the *Eco*RI site of pRK415 to generate the knockout vector and was then sequenced to confirm the presence of the desired gene and check for the integrity of the sequence. The knockout vector was delivered to *S. wittichii* RW1 (wild type) and transferred for several times on 5 ml LB broth as mentioned above. Colonies that were kanamycin resistance and tetracycline sensitive (conducted double homologous recombination which replaced the wild type copy of *icl* with the disrupted copy of *icl* constructed on pRK415) were isolated and confirmed by PCR of the fragment containing *icl* and compared to the wild type.

## Results

### Effect of isocitrate lyase deletion on acetate utilization

**Fig. 1:**
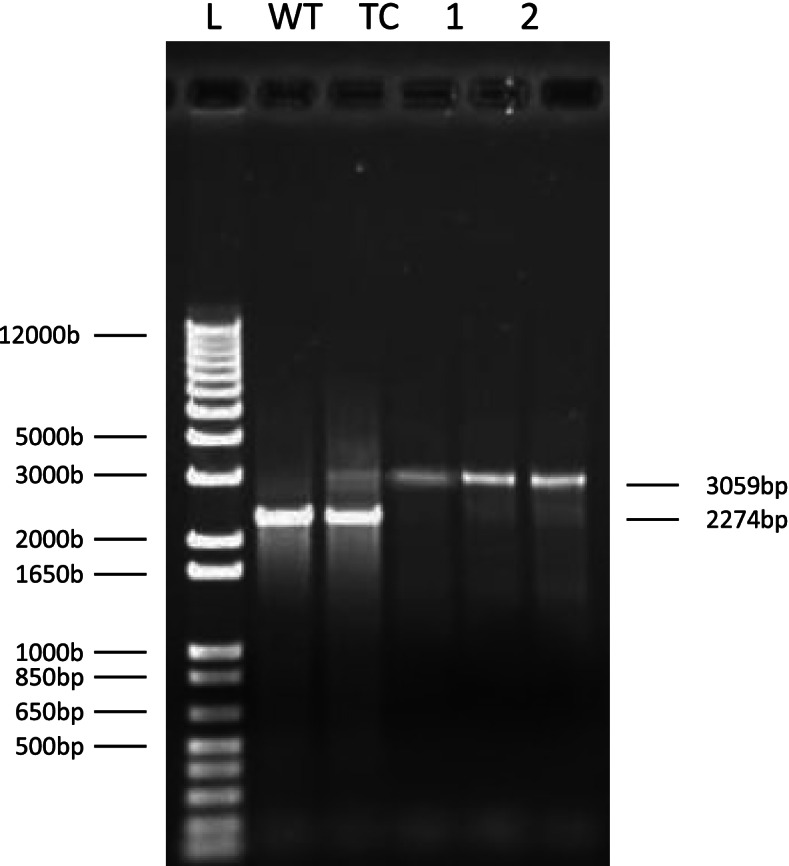
Conformation of the mutant strains by PCR. 1 Kb plus DNA ladder (L). Wild type gene of icl locus (WT). PCR of transconjugants showing both mutated and wild type region of icl (TC). PCR of icl mutants showing the mutated *icl* after double crossover (1,2, and 3)

After several transfers on LB media, several transconjugants were isolated that had experienced double homologous recombination between the wild type copy of *icl* gene and its disrupted copy on the knockout vector. Results were confirmed by PCR using the primers icl-F and icl-R as shown in Fig. 1.

**Fig. 2:**
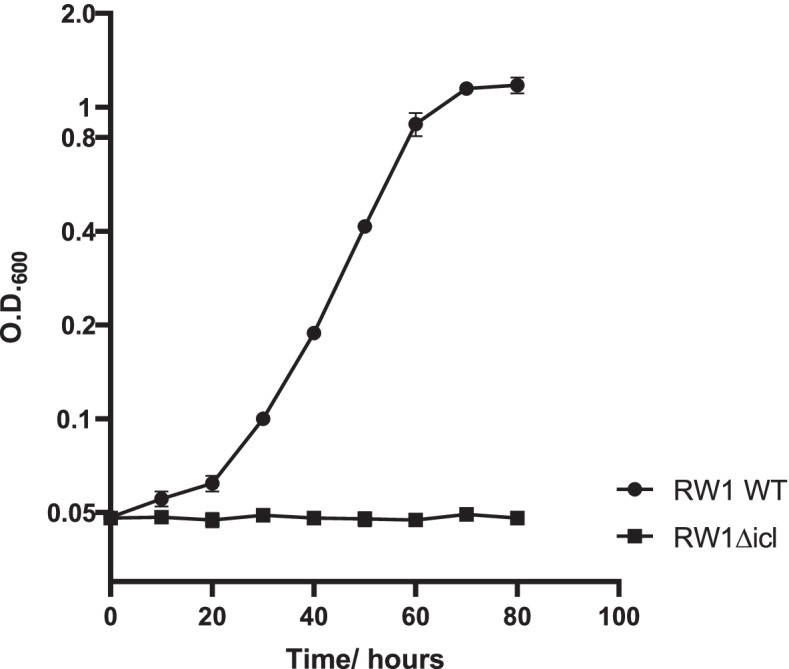
Effect of *icl* deletion on growth of *S. wittichii* RW1 on 10 mM acetate

As expected, deletion of *icl* from *S. wittichi* RW1 abolished this bacterium from growing on acetate as a sole carbon source (Fig. 2). Complementation experiments conducted by transforming RW1Δicl with a pRK415 vector carrying an intact copy of *icl* reverted the transformed knockout strain to its wild-type state (data not shown).

### Effect of isocitrate lyase deletion on DBF and DD degradation

**Fig. 3:**
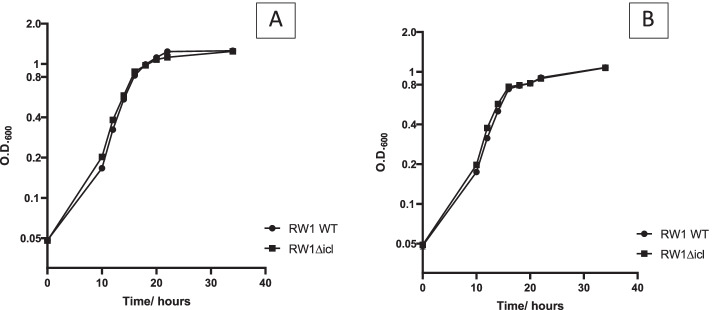
Effect of *icl* deletion on growth of *S. wittichii* RW1 on DBF (**A**) and DD (**B**). DBF (3 mM) and DD (3 mM) were added as crystals in MSB medium as a sole carbon source

Growth curve experiments on DBF and DD were done for *S. wittichii* RW1Δicl and compared to the growth of the wild type strain. Fortunately, no differences in growth rates was observed between the knockout strains and the wild type (Fig. 3) indicating that *icl* deletion has no effect on the capability of *S. wittichi* RW1 to metabolize DBF and DD and this strain could be used as a suitable candidate to remediate DD and DBF polluted sites that are rich in acetate.

## Discussion

**Fig. 4:**
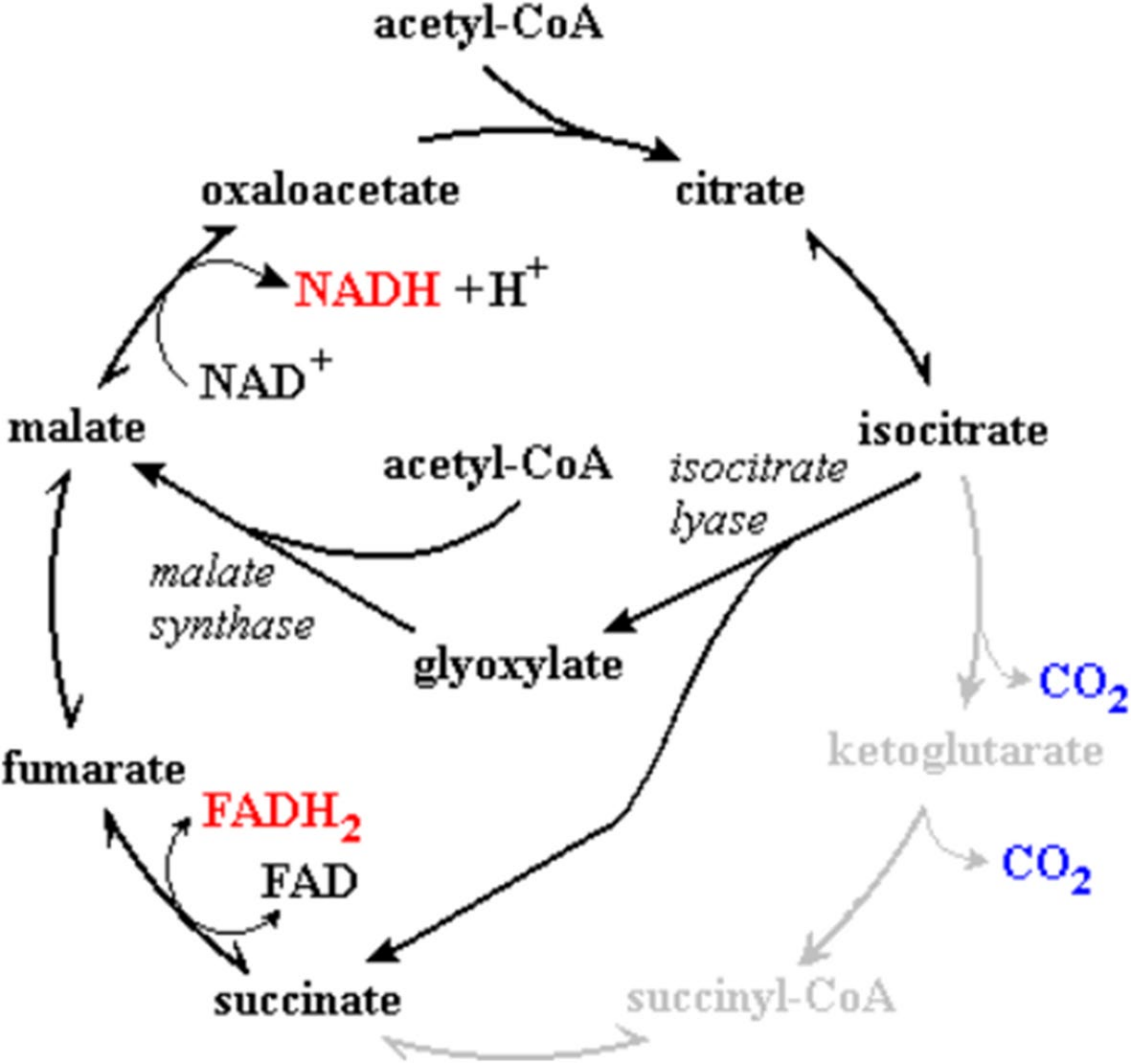
Glyoxylate shunt pathway

Isocitrate lyase along with malate synthase are two key enzymes in bacteria for the glyoxylate shunt pathway. Isocitrate lyase catalyzes the split of isocitrate to glyoxylate and succinate while the former is converted to malate via malate synthase (Fig. 4). The glyoxylate shunt enables bacteria to grow on acetate as it bypasses the loss of the two carbons in acetate as carbon dioxide in the TCA cycle [18].

Deleting *icl* from RW1 lost the ability of RW1Δicl to utilize acetate as a sole carbon source while the wild type phenotype was reverted when an intact copy of *icl* was introduced to RW1Δicl. These results all together identify the importance of *icl* in the metabolism of acetate and possibly all other two carbon containing molecules and provides evidence for the presence of a single copy of *icl* in this bacterium. In addition, complementation experiments verify that the phenotype detected was due to *icl* deletion and not to a polar effect of downstream genes. No previous studies have deleted *icl* from *S. wittichii* RW1; however, site-directed mutagenesis of this gene in *E. coli* have showed the importance of four histidine residues. The highest effective mutation was seen with H-184. Substitution of H-184 to lysine, arginine, or leucine lead to total loss of enzyme activity while the mutated protein with H-184 substituted with glycine retained 28% of its activity. Protein gel electrophoresis of these mutated proteins showed weak assembly of the Icl subunits into the tetrameric active protein [6].

**Fig. 5:**
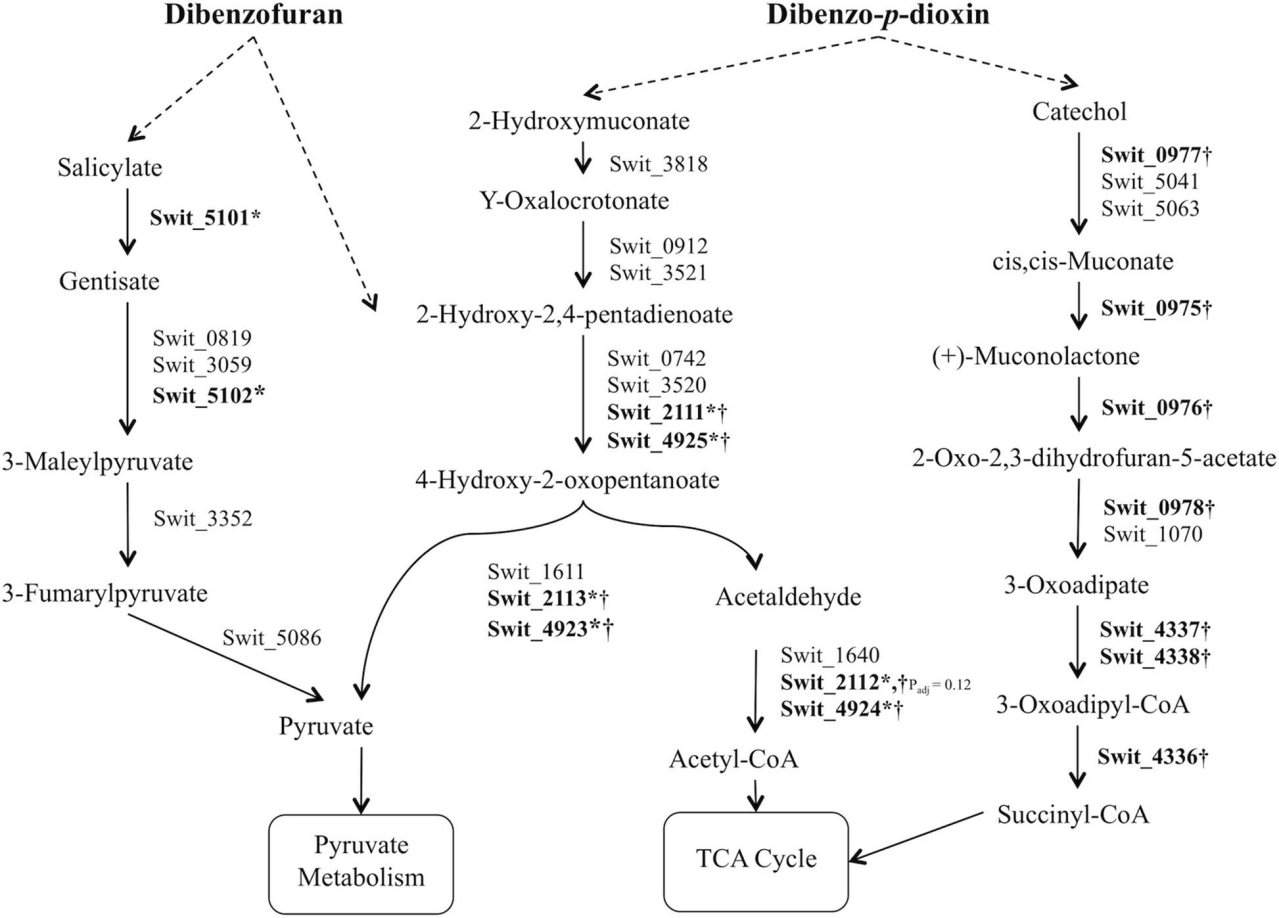
The lower catabolic pathway for DBF and DD degradation. Upregulated genes are indicated in bold, (*) with DBF and (†) with DD

Due to the fact that *icl* deletion had no effect on the degradation of DBF and DD, our results shows that this mutant strain is a suitable candidate for remediating such toxic aromatics from environments that are rich in acetate. Interestingly, acetate is one of the intermediates formed from the downstream degradation pathway of DBF and DD and represents 2 out of 12 carbon atoms of their structure (Fig. 5). However, blocking the mutant from using acetate had no effect on its growth rate on these aromatics the reason is probably due to the use of acetate formed from the degradation pathways in the synthesis of fatty acid and other essential building blocks in the cell. *S. wittichii* RW1 has shown to manage gene regulation for DD and DBF degradation according to its surroundings. A recent study showed that this bacterium adapted the changes in DD availability when DD was absorbed to smectite clay mineral saponite by up-regulating genes that were thought to be involved in the uptake and chemotaxis of DD [2]. In *E. coli* acetate can be metabolized to acetyl-coA through two major pathways: the reversible Pta-AckA pathway and the irreversible acetyl-coA synthetase pathway. Feeding *E. coli* growing in log phase with ^13^C- acetate resulted in the detection of labeled metabolites not only of TCA intermediates but from all central carbon metabolites of carbohydrates [7].

A different study showed that effects of adding acetate decreased the degradation rate of several pharmaceuticals due to feedback inhibition of acetate concentrations which comes in an agreement with our study [14]. These results support our idea for acetate flow and indicates that acetate can be channeled through different pathways in bacteria as expected. In addition, environmental bacteria including *S. wittichii* RW1 when present in the environment faces carbon-limited conditions which has been shown to induce the expression of polyaromatic hydrocarbon degrading genes [23]. As a results, our mutant strain that is incapable of using acetate will be continually facing carbon-limited conditions and will have improved DD and DBF degrading capabilities. However, the presence of other acetate metabolizers in the environment might be considered as a limitation of this study. Future studies are needed to confirm our work along with growth curve experiments showing the disappearance of DD and DBF when acetate is present or absent. Recent studies have incorporated the use of omics in biodegradation studies for better understanding of how microbes interact with each other in the environment [15]. Such experiments may be useful to identify how the presence of other organisms in contaminated area may affect DD and DBF degradation.

## Conclusion

This research was successfully capable of producing an engineered strain of *S. wittichii* RW1 that has a metabolic block in the glyoxylate shunt through deletion of isocitrate lyase gene. The mutant RW1Δicl was no longer capable of growing on acetate as sole carbon source compared to the wild type. However, RW1Δicl capability to metabolize DBF and DD was not affected. This strain will be a perfect candidate to metabolize such aromatics in contaminated sites rich with acetate.

## Data Availability

Not applicable.

## Abbreviations

DBF: Dibenzofuran
DD: Dibenzodioxin
icl: Isocitrate lyase
MSB: Mineral salt medium
Rpm: Round per minute
LB: Luria-Bertani
WT: Wild type
TC: Transconjugant
TCA: Tricarboxylic acid cycle
